# O03 Continuation of golimumab (anti-TNF) in a patient with SpA and low-risk prostate cancer, what is the right decision?

**DOI:** 10.1093/rap/rkab067.002

**Published:** 2021-10-19

**Authors:** Amelia Holloway, Catherine Mathews

**Affiliations:** Queen Elizabeth Hospital, London, United Kingdom

## Abstract

**Case report - Introduction:**

Golimumab is an anti-TNF alpha drug used in the treatment of inflammatory arthritis including spondyloarthritis (SpA). The introduction of this drug class has revolutionised the treatment of SpA over the last 20 years with significantly improved patient outcomes.

Despite their treatment benefits multiple adverse effects of TNF-alpha inhibition have been reported through clinical trials including a possible increased risk of malignancy.

We describe a case of a patient with known ankylosing spondylitis (AS) on golimumab who was diagnosed with low-grade prostate carcinoma and discuss the factors taken into consideration in guiding our decision-making process regarding ongoing treatment.

**Case report - Case description:**

A 57-year-old gentleman with known AS presented to the rheumatology clinic for routine review. His AS was well controlled, and he had been taking golimumab for the past 3 years. Upon review he was in clinical remission with a CRP <1 and ESR 5. Prior to the initiation of anti-TNF therapy his disease had been poorly controlled. However, following commencement his symptoms had significantly improved and he was able to work as a professional sports coach whilst bringing up a young family.

On review he had recently been diagnosed with low-risk cancer of the prostate by his urologist. A prostate biopsy found Gleason 3 + 3 adenocarcinoma involving 2 out of 22 cores on each side, with a prostate specific antigen (PSA) of 3.95ng/ml. An MRI had shown chronic prostatitis. He was in the lowest risk category of grade group 1 prostate cancer and no treatment for his prostate cancer was indicated. The plan from his urology team was active surveillance with PSA monitoring.

Whilst being investigated for possible malignancy his golimumab had been held for six months and during this period he had a significant flare in symptoms. He experienced severe back pain that forced him to stop working. Following his prostate cancer diagnosis, golimumab was restarted by his urologist with a subsequent improvement in his AS symptoms.

To guide ongoing treatment his case was reviewed in the local biologics multi-disciplinary team meeting, alongside close communication with his urologist.  The patient was informed of the risks of continuing golimumab in relation to his malignancy. Despite this he was reluctant to stop anti-TNF therapy or switch to another treatment, citing concerns about the impact it might have on his symptoms and ability to work.

**Case report - Discussion:**

This case highlights the complexities involved in the management of a patient on anti-TNF therapy, who receives a diagnosis of malignancy, particularly when the diagnosis is classed as low risk. Traditionally anti-TNF therapy was contraindicated for patients with a history of a solid organ tumour within the previous five years. The British Society of Rheumatology (BSR) guidelines recommends that patients should be advised that there is no conclusive evidence for an increased risk of solid organ tumours but that on-going vigilance is required.

A holistic patient-centred approach needs to be taken in these contexts, and consideration of cases on an individual basis is needed. Inter-disciplinary and multi-speciality team input, with the effective use of a biologics MDT, is crucial. The patient was understandably reluctant to stop his treatment due to the significant impact this may have on his quality of life.

On liaison with his urologist his prostate cancer was in the lowest risk category with 99% 5-year survival rates with low risk of disease progression or spread.

Evidence in this field to date has been conflicting and studies have predominantly focused on the safety of anti-TNFs in rheumatoid arthritis patients. Recent large national registry data has been reassuring. Few studies have looked at the AS and psoriatic arthritis anti-TNF treated population; however, a meta-analysis of RCTs found no evidence of increased incidence of malignancy.

Taking into account his low-risk cancer, the patient’s wishes and clinical evidence in this field we have made to decision to continue anti-TNF treatment for now but with ongoing surveillance for any tumour progression. The patient will undergo urology follow up alongside regular PSA monitoring, and there will be a low threshold to stop or switch treatment in the future

**Case report - Key learning points:**

1. Treatment with anti-TNF therapy in patients with concomitant inflammatory arthritis and malignancy pose a specific challenge that require close co-ordination of care between rheumatologists, oncologists, other relevant teams and may benefit from the use of local biologics MDT to guide ongoing drug therapy.
2. It is important to take a patient centred approach and consider cases on an individual basis taking into account their cancer diagnosis, extent of disease, histology findings and their response to TNF treatment.
3. Patients must always be counselled on the risk of continuing anti-TNF and be involved in the decision-making process.
4. Further research is required to look at the safety of anti-TNF particularly in SpA and if there is an increased risk of malignancy.

